# Morpho-phylogenetic analyses reveal *Elliptolagenoidisporium* gen. nov. and a new species associated with the medicinal plant *Paris* sp. from Guizhou Province, China (*Helotiales*, *Lachnaceae*)

**DOI:** 10.3897/mycokeys.131.182322

**Published:** 2026-04-15

**Authors:** Xiang-Yu Zhang, Min Liu, Jian Ma, Xiao-Fang Chen

**Affiliations:** 1 Bijie Institute of Traditional Chinese Medicine, Bijie, Guizhou 551700, China Bijie Institute of Traditional Chinese Medicine Bijie China; 2 Guizhou Key Laboratory for Germplasm Innovation and Resource-Efficient Utilization of Dao-di Herbs, Guiyang, Guizhou 550025, China Guizhou Key Laboratory for Germplasm Innovation and Resource-Efficient Utilization of Dao-di Herbs Guiyang China; 3 Guizhou Industry Polytechnic College, Guiyang, Guizhou 550008, China Guizhou Industry Polytechnic College Guiyang China; 4 Resource Institute for Chinese & Ethnic Materia Medica, Guizhou University of Traditional Chinese Medicine, Guiyang, Guizhou 550025, China Bijie Medical College Bijie China; 5 Bijie Medical College, Bijie, Guizhou 551700, China Resource Institute for Chinese & Ethnic Materia Medica, Guizhou University of Traditional Chinese Medicine Guiyang China

**Keywords:** Asexual morph, phylogeny, saprobic fungi, *
Sordariomycetes
*, taxonomy

## Abstract

A new genus, *Elliptolagenoidisporium*, is proposed herein to accommodate a novel hyphomycetous fungus, *E. paris*, isolated from dead branches of the medicinal plant *Paris* sp. from Guizhou Province, China. Phylogenetic analyses based on a combined dataset of LSU and ITS sequences robustly support the placement of *Elliptolagenoidisporium* within the family *Lachnaceae (Helotiales)*. Comprehensive morphological descriptions, illustrations, taxonomic notes, and phylogenetic evidence are provided to substantiate the establishment and systematic position of this new genus and species. Currently, *Elliptolagenoidisporium* is known only from its asexual morph, which is characterized by micronematous, mononematous, solitary, erect, simple or branched, septate, straight or flexuous, cylindrical conidiophores; holoblastic, integrated, terminal, cylindrical to lageniform conidiogenous cells; and acrogenous, solitary, septate, acerose, fusiform, lageniform to ellipsoidal, verrucose, guttulate, smooth or echinulate conidia. The discovery of *E. paris* provides valuable insights into the species diversity, ecological functions, and biogeographical distribution of saprobic fungi associated with medicinal plants in karst ecosystems.

## Introduction

Medicinal plants play a critical role in the prevention and treatment of human diseases, owing to their rich repertoire of bioactive compounds that exhibit a wide range of pharmacological activities, including antimicrobial, anti-inflammatory, antioxidant, and anticancer properties (Nalawade et al. 2003; Cole et al. 2007; Schmidt 2017; Rahman et al. 2019). The structural and functional diversity of secondary metabolites derived from medicinal plants, such as alkaloids, flavonoids, terpenoids, and phenolics, provides substantial opportunities for the discovery and development of novel pharmaceuticals and therapeutic agents (Samy and Gopalakrishnakone 2007; Ali et al. 2021; Atanasov et al. 2021). Southwest China, particularly Guizhou Province, is recognized as a major center of traditional Chinese medicine due to its exceptional diversity of medicinal plants (Du et al. 2024). This diversity is largely driven by the region’s distinctive karst landscapes, which create heterogeneous microhabitats and ecological niches that promote speciation, genetic differentiation, and adaptive evolution of both plants and associated microbial communities (Huang et al. 2012; Taylor et al. 2014; Lu et al. 2018; Guo et al. 2020; Shan et al. 2022). In addition to their medicinal value, these plants support complex microbial assemblages, including endophytic, pathogenic, and saprobic fungi, many of which remain undescribed. Recent investigations in Guizhou Province have revealed a substantial number of previously undocumented microfungi associated with medicinal plants, highlighting the region as a hotspot of fungal biodiversity and underscoring its potential for the discovery of novel taxa with ecological, agricultural, and pharmacological significance (Abtahi and Nourani 2017; Helaly et al. 2018; Keshri et al. 2021; Du et al. 2022a, 2022b; Sun et al. 2023, 2025; Wu et al. 2024; Zou et al. 2025). These findings highlight the potential of medicinal plants as valuable reservoirs for uncovering fungal diversity and enhancing our understanding of plant–fungus interactions in ecologically unique habitats.

The family *Lachnaceae (Helotiales)* was established by Raitviir (2004), with *Lachnum* designated as the type genus. Members of *Lachnaceae* are typically characterized by small, cup-shaped apothecia and distinctive hairy or setose marginal structures, which have historically led to considerable taxonomic confusion within the group (Han et al. 2014; Guatimosim et al. 2016; Phookamsak et al. 2019; Visagie et al. 2024; Hongsanan et al. 2025). Early classifications placed many of these taxa within *Hyaloscyphaceae*, a large and morphologically heterogeneous family within the order *Helotiales*. However, subsequent molecular studies have demonstrated that *Hyaloscyphaceaesensu lato* contains multiple distinct evolutionary lineages (Retzius 1779; Karsten 1884; Kirschstein 1938; Baral and Krieglsteiner 1985). A comprehensive phylogenetic reassessment by Han et al. (2014) demonstrated that *Lachnum* and morphologically allied genera form a well-supported, monophyletic clade within *Hyaloscyphaceaesensu lato*, thereby validating *Lachnaceae* as an independent family. Currently, 17 genera are accepted within the family *Lachnaceae*, including *Albotricha*, *Asperopilum*, *Belonidium*, *Brunnipila*, *Cadophorella*, *Capitotricha*, *Dasyscyphella*, *Erioscyphella*, *Incrucipulum*, *Lachnellula*, *Lachnopsis*, *Lachnum*, *Neodasyscypha*, *Perrotia*, *Proliferodiscus*, *Tubolachnum*, and *Velebitea* (Raitviir 2004; Han et al. 2014; Guatimosim et al. 2016; Phookamsak et al. 2019; Hyde et al. 2024; Visagie et al. 2024; Hongsanan et al. 2025).

During a survey of saprobic fungi associated with medicinal plants in Guizhou Province, China, we recovered a previously undescribed hyphomycetous taxon, assignable to the family *Lachnaceae* based on morphological and molecular evidence. Phylogenetic analyses of combined LSU and ITS sequence datasets revealed that the newly collected isolate forms a well-supported and phylogenetically distinct clade within *Lachnaceae*, separate from all currently known genera in the family. By integrating detailed morphological examinations of the conidiophores, conidiogenous cells, and conidial characteristics with molecular phylogenetic evidence based on combined LSU and ITS datasets, we propose a new hyphomycetous genus, *Elliptolagenoidisporium*, to accommodate this distinct lineage, *E. paris*. The present study provides a comprehensive taxonomic treatment of the new taxon, including an updated phylogeny of *Lachnaceae*, detailed morphological descriptions, illustrations, and comparative assessments with phenotypically similar genera. Our findings not only contribute to the growing inventory of saprobic fungi associated with medicinal plants in Southwest China but also underscore the ecological significance of karst-region flora as reservoirs of fungal diversity. The discovery of this new lineage enriches the taxonomic framework of *Lachnaceae* and advances our understanding of fungal evolution, host associations, and niche specialization within this ecologically diverse family.

## Materials and methods

### Sample collection, isolation, and morphological studies

Fresh samples were collected from the medicinal plant *Paris* sp. in Datian Yi, Miao, and Buyi Ethnic Town, Jinsha County, Bijie City, Guizhou Province, China, on 25 March 2025. Specimens were carefully stored in zip-lock plastic bags, meticulously labeled using a marker pen (Rathnayaka et al. 2025), and examined under a stereomicroscope (SMZ 745, Nikon, Japan) in the laboratory. Collection, examination, and isolation procedures followed the protocols described by Senanayake et al. (2020) and Rathnayaka et al. (2025). Micro-morphological characters were captured using a Nikon EOS 90D digital camera attached to an ECLIPSE Ni compound microscope (Nikon, Japan). Morphological measurements were performed using the Tarosoft (R) Image Framework software (version IFW 0.97), while photomicrographs were constructed employing Adobe Photoshop 2019 software (Adobe Systems, USA).

Following the morphological examination, the specimens were deposited at the herbarium of the Guizhou Academy of Agricultural Sciences (**GZAAS**), Guiyang, China. In addition, ex-type living cultures were preserved in the Guizhou Culture Collection (**GZCC**). Species recognition and the justification for establishing new species followed the guidelines proposed by Chethana et al. (2021).

### DNA extraction, PCR amplification, and sequencing

Mycelium freshly scraped from living cultures was transferred to 1.5 mL microcentrifuge tubes and stored at –20 °C. Genomic DNA was extracted using a DNA extraction kit from Sangon Biotech (Shanghai) Co., Ltd., China. Polymerase chain reaction (PCR) was performed to amplify the DNA regions using established primer pairs: LR0R/LR5 for LSU (Vilgalys and Hester 1990; Cubeta et al. 1991) and ITS5/ITS4 for ITS (White et al. 1990). PCR was performed in a 50 μL reaction volume containing 2 μL of DNA template, 2 μL of forward primer, 2 μL of reverse primer, 25 μL of 2× Taq PCR Master Mix, and 19 μL of double-distilled water. The thermal cycling conditions for both LSU and ITS regions consisted of an initial denaturation at 94 °C for 3 min, followed by 40 cycles of denaturation at 94 °C for 45 s, annealing at 56 °C for 50 s, and elongation at 72 °C for 1 min, with a final extension at 72 °C for 10 min. PCR products were verified on 1% agarose gels before submission to Sangon Biotech (Shanghai) Co., Ltd., China, for sequencing.

### Phylogenetic analyses

Forward and reverse sequences from the newly generated sequence were assembled using Contig Express v3.0.0. Additional reference sequences used in this study (Table 1) were downloaded from the NCBI GenBank database (https://blast.ncbi.nlm.nih.gov/Blast.cgi). Individual sequences were aligned using the online version of MAFFT v. 7 (https://mafft.cbrc.jp/alignment/server/index.html) (Katoh et al. 2017). The LSU and ITS alignments were trimmed with trimAl v1.2rev59 (Capella-Gutiérrez et al. 2009) and subsequently merged with SequenceMatrix v1.7.8 (Vaidya et al. 2011).

**Table 1.** Taxa used in this study and their GenBank accession numbers for LSU and ITS sequence data.

Taxon	Strain	GenBank accessions	
		LSU	ITS
*Acephala applanata*	CBS 109321	KF951051	NR_119482
*Arachnopeziza aurata*	KUS-F52038	JN086696	JN033393
*A. delicatula*	TNS-F12770	JN086736	JN033433
*A. obtusipila*	TNS-F12768	JN086746	JN033445
*Botrytis cinerea*	OSC 100012	AY544651	DQ491491
*Brunnipila fuscescens*	KUS F52031	JN086695	JN033392
*Bulgaria inquinans*	AFTOL-ID 916	DQ470960	N/A
*Cadophorella faginea*	CPC 45667^T^	PP872405	PP872394
*Capitotricha filiformis*	MFLU 15-2784^T^	N/A	MK584992
*C. pterosparti*	E.R.D. 6236^T^	MN044100	N/A
*Cenangium ferruginosum*	KL390	KX090840	LT158471
*Chaetopsis canovae*	CBS 150.58	MH869271	MH857732
*Chlorencoelia torta*	KUS-F52256	JN086703	JN033400
*Chlorociboria glauca*	KL238	KX090821	N/A
*Cordierites guianensis*	192	EU107270	N/A
*Dasyscyphella chrysotexta*	CNF 2/100072^T^	MH886411	MH886407
*Diplocarpa bloxami*	KL317	KX090834	N/A
***Elliptolagenoidisporium paris***	**GZCC 25-27590^T^**	**PX848715**	**PX848701**
***E. paris***	**GZCC 25-27591**	**PX848716**	**PX848702**
*Erioscyphella gelangheica*	HKAS 135695^T^	PQ349780	PQ349788
*E. tengyueica*	HKAS 135693^T^	PQ349782	PQ349790
Hyaloscypha albohyalina var. albohyalina	TNS-F17137	JN086734	JN033431
*H. hepaticicola*	M. Kukkonen 37	EU940150	EU940226
*H. vitreola*	T. Laukka 229	EU940156	EU940232
*Hymenoscyphus albidoides*	HMAS 264140^T^	NG_059508	NR_154903
*Hy. aurantiacus*	HMAS 264143^T^	NG_059509	NR_154907
*Hy. caudatus*	KUS-F52291	JN086705	JN033402
*Hy. fructigenus*	M.L. & P. Heinonen 708	EU940157	EU940233
*Hy. immutabilis*	HMAS 287040	OQ534463	OQ534178
*Hy. yui*	HMAS 266595^T^	NG_241867	NR_189343
*Incrucipulum capitatum*	TNS-F81420	LC424954	LC424838
*I. foliicola*	FC 6879^T^	LC438584	LC438567
*I. hakonechloae-macrae*	TNS-F81512^T^	LC438581	LC438564
*I. pseudosulphurellum*	TNS-F81441^T^	LC438587	LC438570
*I. radiatum*	FC 2283	AB481322	AB481261
*I. sulphurellum*	SBRH 859	KX501131	KX501128
*Lachnellula calyciformis*	CBS 189.66	KC492973	MH858771
*Lachnopsis catarinensis*	CPC 24723^T^	KU597761	NR_154125
*L. dicksoniae*	CPC 24742	KU597763	KU597796
*Lachnum virgineum*	OSC 100002	AY544646	DQ491485
*Leotia lubrica*	AFTOL-ID 1	AY544644	DQ491484
*L. viscosa*	468E2CE	AF113737	N/A
*Leuconeurospora pulcherrima*	AFTOL-ID 1397	FJ176884	N/A
*Microglossum rufum*	AFTOL-ID 1292	DQ470981	N/A
*Mollisia cinerea*	OSC 100029	DQ470942	DQ491498
*Monilinia laxa*	OSC 100063	AY544670	N/A
*Phacidium pseudophacidioides*	CBS 590.69^T^	KJ663894	KJ663853
*Potebniamyces pyri*	AFTOL-ID 744	DQ470949	DQ491510
*Proliferodiscus chiangraiensis*	MFLU 16-0588^T^	MK591985	NR_164304
*P. ingens*	CBS 145519^T^	MN232929	MN232961
*Pseudeurotium hygrophilum*	229	JQ780654	JQ780653
*Ps. zonatum*	AFTOL-ID 1912	DQ470988	N/A
*Psilachnum staphyleae*	KUS-F52105	JN086699	JN033396
*Rhymbocarpus fuscoatrae*	Ertz 16200	KJ559571	KJ559549
*Rutstroemia firma*	KL292	KX090832	LT158450
*R. luteovirescens*	KL217	KX090814	LT158431
*Sclerotinia sclerotiorum*	CBS 499.50	DQ470965	N/A
*Togniniella acerosa*	PDD 81432	AY761076	N/A
*T. acerosa*	CBS 113726^T^	NG_057734	NR_135947
*Trichopeziza aff. mollissima*	G.M. 2023-06-10.1 #2342	PP076721	PP076721
*Vibrissea truncorum*	CBS 258.91	FJ176874	N/A

Note: “^T^” indicates ex-type strains. The newly generated sequence is in red bold. “N/A” indicates the unavailable data in GenBank.

Phylogenetic analyses were conducted using RAxML-HPC v.8 on XSEDE (8.2.12), with a GTRGAMMA model and rapid bootstrap analysis followed by 1000 bootstrap replicates (Stamatakis 2014). Bayesian inference (BI) analysis was performed using MrBayes on XSEDE (3.2.7a) via CIPRES (Swofford 2002; Miller et al. 2010; Ronquist et al. 2012). The aligned FASTA file was converted to NEXUS format using AliView (Daniel et al. 2010). The best-fit evolutionary model for the individual dataset was determined using MrModeltest v. 2.3.10 (Nylander 2004). The best-fitting nucleotide substitution model selected for the Bayesian inference (BI) analysis was GTR+I+G for both the LSU and ITS datasets. Posterior probabilities (PP) were determined based on Bayesian Markov chain Monte Carlo (BMCMC) sampling (Huelsenbeck and Ronquist 2001). Two simultaneous Markov chains were run for 10,000,000 generations, and trees were sampled every 1,000^th^ generation. The burn-in phase was set at 25%, and the remaining trees were used for calculating posterior probabilities (PP).

Phylogenetic trees were visualized using FigTree version 1.4.0 and further edited using Adobe Photoshop 2019 program (Adobe Systems, USA) and Adobe Illustrator version 51.1052.0.0 (Adobe Inc., San Jose, California, USA).

### Phylogenetic analysis results

The newly analyzed taxa were resolved within the *Elliptolagenoidisporium* clade (*Lachnaceae*, *Helotiales*), based on combined phylogenetic analyses of LSU and ITS sequence data. The dataset, consisting of DNA sequences from 61 taxa with a total alignment length of 1,482 characters (including gaps; LSU: 1–908 bp; ITS: 909–1,482 bp), was used for phylogenetic analyses. Phylogenetic analyses were conducted using maximum likelihood (ML) and Bayesian inference (BI) analyses, with *Togniniella acerosa* (CBS 113726) and *T. acerosa* (PDD 81432) designated as outgroup taxa. Base frequencies and rates were A = 0.248800, C = 0.220859, G = 0.286415, and T = 0.243927; and substitution rates were AC = 1.505026, AG = 2.899904, AT = 1.665057, CG = 1.105947, CT = 7.297923, and GT = 1.000000. The distribution shape parameter (*α*) equaled 0.294539.

**Figure 1.**
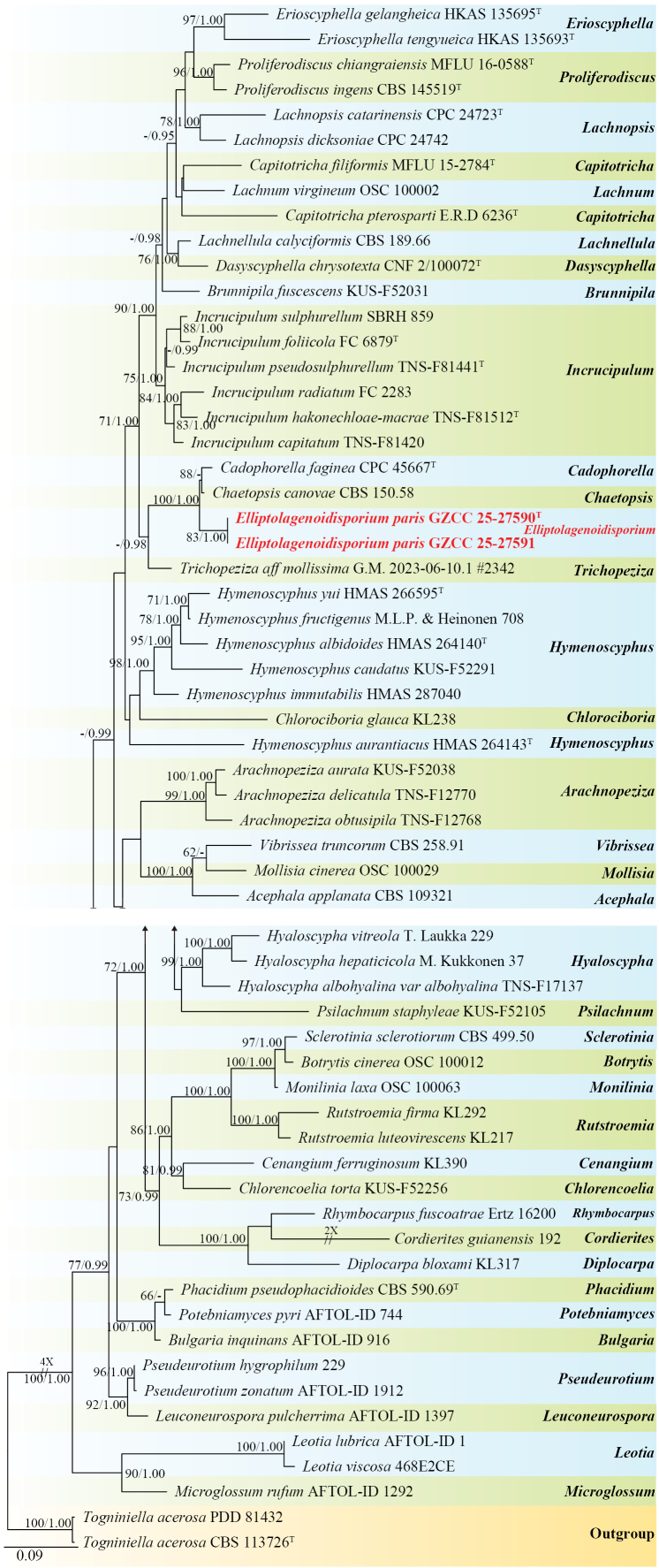
Phylogenetic analysis of *Elliptolagenoidisporium* was conducted using RAxML-based maximum likelihood analyses of a combined LSU and ITS DNA sequence dataset. Bootstrap support values for maximum likelihood (ML) equal to or greater than 60% (ML left) and Bayesian posterior probabilities (BI right) equal to or greater than 0.95 are shown above the nodes. Hyphen (“-”) indicates a value lower than 60% for ML and a posterior probability lower than 0.95 for Bayesian inference. The tree is rooted with *Togniniella acerosa* (CBS 113726) and *T. acerosa* (PDD 81432). Ex-type strains are denoted with “^T,^” and newly obtained isolates are shown in bold red fonts.

Based on the combined LSU and ITS phylogenetic tree (Fig. 1), our collections represent a new genus and a novel species within the family *Lachnaceae (Helotiales)*. The isolates GZCC 25-27590 and GZCC 25-27591 formed a sister clade to the clade comprising *Cadophorella faginea* (CPC 45667) and *Chaetopsis canovae* (CBS 150.58) with 100% ML/1.00 BI bootstrap support.

## Taxonomy

### Elliptolagenoidisporium

Taxon classificationFungiHelotialesLachnaceae

X.Y. Zhang & X.F. Chen gen. nov.

978AB6C1-31DD-556D-BA88-C7E56EDDC947

904729

#### Etymology.

“*Elliptolagenoidisporium*” refers to the elliptic-lageniform conidial characteristics of this genus.

#### Description.

*Saprobic* on dead branches of *Paris* sp. **Sexual morph**: Undetermined. **Asexual morph**: Hyphomycetous. ***Colonies*** superficial, effuse, scattered or gregarious, velvety, pale brown to brown. ***Mycelium*** partly superficial, partly immersed, consisting of branched, septate, smooth-walled, hyaline to pale brown hyphae. ***Conidiophores*** micronematous, mononematous, solitary, erect, simple or branched, septate, straight or flexuous, cylindrical, subhyaline to pale brown, sometimes slightly swollen at the base, smooth-walled. ***Conidiogenous cells*** holoblastic, integrated, terminal or lateral, cylindrical to lageniform, subhyaline to pale brown, smooth-walled. ***Conidia*** acrogenous, solitary, 1–2-septate, acerose, fusiform, lageniform to ellipsoidal, verrucose, guttulate, sometimes slightly constricted at the septum, apex rounded, base truncate or slightly attenuated, subhyaline to pale brown, smooth or echinulate.

#### Type species.

*Elliptolagenoidisporium paris* X.Y. Zhang & X.F. Chen

#### Notes.

Morphologically, *Elliptolagenoidisporium* can be readily distinguished from other genera within *Lachnaceae (Helotiales)* by its micronematous, simple or branched conidiophores; holoblastic, cylindrical to lageniform conidiogenous cells; and 1–2-septate, acerose, fusiform, lageniform to ellipsoidal conidia, which may be smooth or echinulate. Phylogenetic analyses further indicate that *Elliptolagenoidisporium* forms a well-supported, distinct subclade within *Lachnaceae*, corroborating its recognition as a separate genus. Based on both molecular evidence and its distinctive fusiform, lageniform to ellipsoidal conidial morphology, we herein establish the genus *Elliptolagenoidisporium* and introduce a novel species, *E. paris*, which is designated as the type species.

### Elliptolagenoidisporium paris

Taxon classificationFungiHelotialesLachnaceae

X.Y. Zhang & X.F. Chen sp. nov.

107503E1-8D78-5425-834D-AC6E5CDB9275

904730

Fig. 2

#### Etymology.

“*paris*’’ refers to the host medicinal plant, *Paris*.

**Figure 2.**
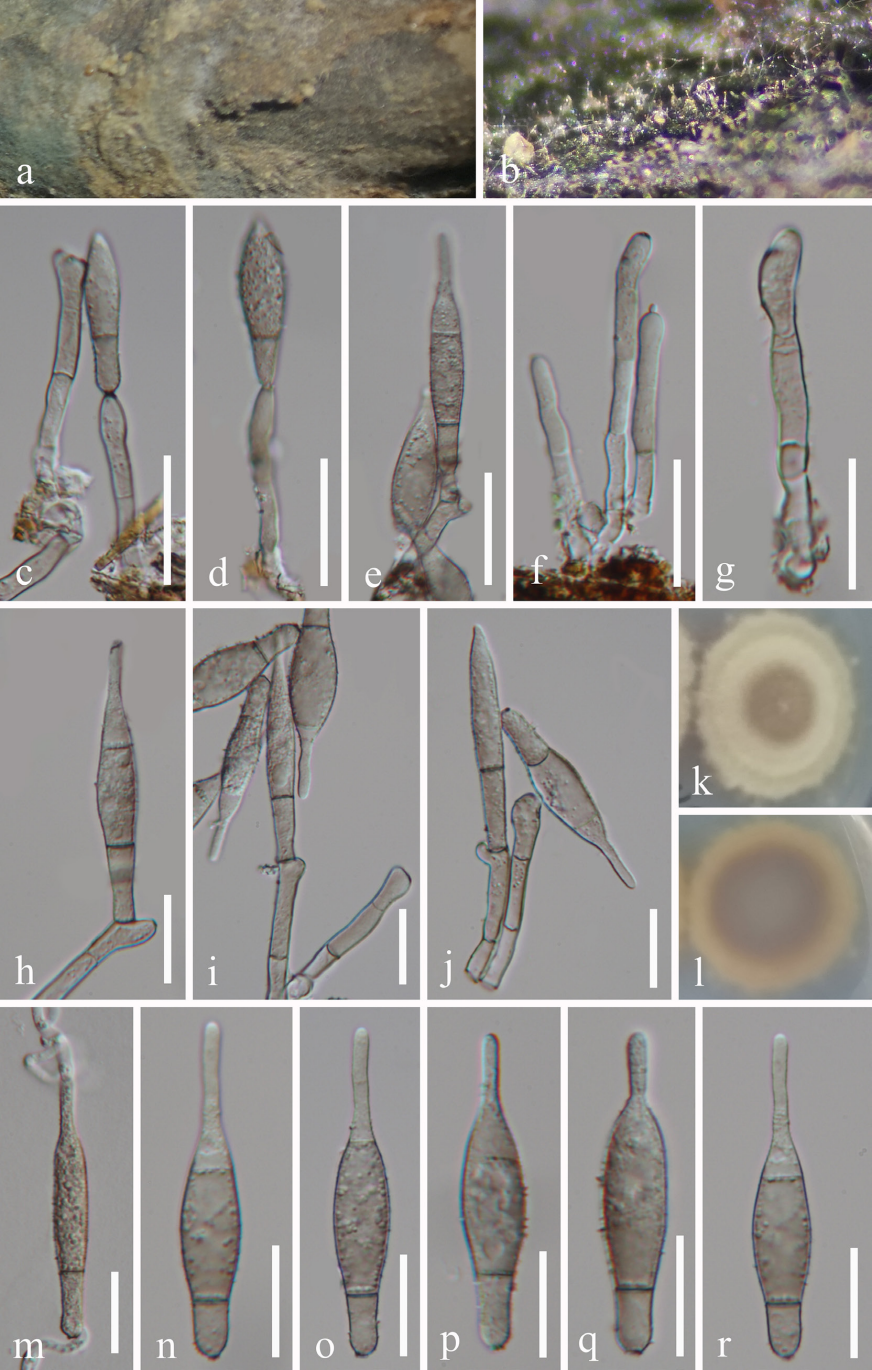
*Elliptolagenoidisporium paris* (GZAAS 25-0781, holotype). **a, b**. Colonies on the host surface; **c–e**. Conidiophores, conidiogenous cells, and conidia; **f, g**. Conidiophores and conidiogenous cells; **h–j**. Conidiogenous cells with attached conidia; **k, l**. Colonies on PDA from above and below after 33 days of incubation at room temperature; **m**. Germinated conidium; **n–r**. Conidia. Scale bars: 30 μm (**c–f**); 20 μm (**g–j, m–r**).

#### Holotype.

GZAAS 25-0781

#### Description.

*Saprobic* on dead branches of *Paris* sp. **Sexual morph**: Undetermined. **Asexual morph**: Hyphomycetous. ***Colonies*** superficial, effuse, scattered or gregarious, velvety, pale brown to brown. ***Mycelium*** partly superficial, partly immersed, consisting of branched, septate, smooth-walled, hyaline to pale brown hyphae. ***Conidiophores*** 31–75 × 4.5–6 μm (x̄ = 47.2 × 5.4 μm, *n* = 20), micronematous, mononematous, solitary, erect, simple or branched, septate, straight or flexuous, cylindrical, subhyaline to pale brown, sometimes slightly swollen at the base, smooth-walled. ***Conidiogenous cells*** 8–26 × 4–6 μm (x̄ = 19 × 5.2 μm, *n* = 25), holoblastic, integrated, terminal or lateral, cylindrical to lageniform, subhyaline to pale brown, smooth-walled. ***Conidia*** 37.5–63 × 8.5–11.5 μm (x̄ = 53.2× 10.4 μm, *n* = 30), acrogenous, solitary, 1–2-septate, acerose, fusiform, lageniform to ellipsoidal, verrucose, guttulate, sometimes slightly constricted at the septum, apex rounded, base truncate or slightly attenuated, subhyaline to pale brown, smooth or echinulate.

#### Culture characteristics.

Conidia germinated on PDA within 12 hours, producing germ tubes from both the apex and base. Colonies on PDA are circular with a raised surface and entire margin, reaching 32 cm in diameter after 33 days at room temperature (approximately 25 °C), greyish-green to pale brown; the reverse pale brown to reddish brown.

#### Material examined.

China • Guizhou Province, Bijie City, Jinsha County, Datian Yi, Miao and Buyi Ethnic Town, on dead branches of *Paris* sp., 25 March 2025, Xiang-Yu Zhang & Xiao-Fang Chen, ZL1 (GZAAS 25-0781, holotype), ex-type living cultures GZCC 25-27590; *ibid*., ZL12 (GZAAS 25-0782, paratype), living culture GZCC 25-27591.

#### Notes.

In the phylogenetic tree, our isolates *Elliptolagenoidisporium paris* (GZCC 25-27590 (ex-type) and GZCC 25-27591) formed a sister clade to *Cadophorella faginea* (CPC 45667) and *Chaetopsis canovae* (CBS 150.58) with 100% ML/1.00 BI bootstrap support (Fig. 1). However, pairwise sequence comparison revealed that *E. paris* (GZCC 25-27590) and *Ca. faginea* (CPC 45667) show 38/471 bp differences in ITS (8.1%, including five gaps) and 11/863 bp differences in LSU (1.3%, including one gap). In addition, our isolate (GZCC 25-27590) differs from *Ch. canovae* (CBS 150.58) by 33/498 bp (6.6%, including five gaps) in ITS and 10/847 bp (1.2%, without gaps) in LSU. Morphologically, *E. paris* (GZAAS 25-0781) can be distinguished from *Ca. faginea* (HPC 4129) by its conidiogenous cells, which are micronematous and cylindrical, whereas those of *Ca. faginea* are monophialidic and subcylindrical to subulate, as well as by its conidia, which are fusiform to lageniform or ellipsoidal, in contrast to the hyaline to olivaceous, obovoid conidia of *Ca. faginea* (Visagie et al. 2024). Moreover, Vu et al. (2019) provided molecular data for *Chaetopsis canovae*; however, no detailed morphological description of the species was provided. However, *Elliptolagenoidisporium paris* differs from the type species of *Chaetopsis* in having holoblastic conidiogenous cells (vs. polyphialidic) and fusiform to lageniform or ellipsoidal conidia (vs. cylindrical) (DiCosmo et al. 1983). Therefore, based on stable, independent branching and incompatible morphological combinations, we propose that our collections (GZCC 25-27590 and GZCC 25-27591) represent a new genus, *Elliptolagenoidisporium*, to accommodate the new species *E. paris*.

## Discussion

Currently, seventeen genera are recognized within the family *Lachnaceae*, of which sixteen are cup fungi (Raitviir 2004; Han et al. 2014; Guatimosim et al. 2016; Phookamsak et al. 2019; Hyde et al. 2024; Visagie et al. 2024; Hongsanan et al. 2025; this study). Among them, seven genera, viz. *Albotricha*, *Asperopilum*, *Belonidium*, *Neodasyscypha*, *Perrotia*, and *Tubolachnum*, remain without molecular data (Durieu de Maisonneuve 1848; Tranzschel 1899; Boudier 1901; Velenovský 1934; Raitviir 1970; Haines and Dumont 1983; Spooner 1987). In this study, we introduce a new hyphomycetous genus, *Elliptolagenoidisporium*, isolated from the medicinal plant *Paris* sp., representing the second asexual genus reported within *Lachnaceae* (Han et al. 2014; Guatimosim et al. 2016; Phookamsak et al. 2019; Visagie et al. 2024; Hongsanan et al. 2025). This genus is distinguished by acerose, fusiform, lageniform to ellipsoidal conidia that are smooth or echinulate, clearly separating it from *Cadophorella*, the first hyphomycetous genus described in the family, which produces obovoid conidia (Visagie et al. 2024). This discovery expands the known morphological diversity and taxonomic breadth of *Lachnaceae*.

Research on fungi in the family *Lachnaceae* has long focused primarily on morphological descriptions (Han et al. 2014; Guatimosim et al. 2016; Phookamsak et al. 2019; Visagie et al. 2024; Hongsanan et al. 2025). Despite these efforts, molecular data remain limited for most species, hindering robust phylogenetic reconstruction and accurate delimitation of genera within the family (Han et al. 2014; Visagie et al. 2024; Hongsanan et al. 2025). In recent years, interest in plant-associated fungi, particularly endophytes, has grown due to their ecological roles and potential biotechnological applications (Sun et al. 2013). For instance, Sun et al. (2013) screened endophytic isolates from *Paris* for antibacterial activity and identified seven strains that inhibited *Staphylococcus aureus*, highlighting their potential as sources of novel antimicrobial compounds. Despite the critical roles of saprobic fungi in nutrient cycling, organic matter decomposition, and ecosystem maintenance, studies focusing on their presence in *Paris* and other medicinal plants remain limited. The karst regions of Southwest China, characterized by unique ecological conditions and diverse medicinal flora, present an important opportunity to investigate the diversity of saprobic fungi associated with these plants. Such research addresses a significant knowledge gap and may lead to the discovery of previously undescribed taxa. These investigations advance understanding of plant–fungus interactions, fungal biodiversity, and the potential applications of saprobic fungi in medicine and biotechnology.

The identification of *Elliptolagenoidisporium paris* expands the known morphological diversity within *Lachnaceae* and deepens understanding of saprobic fungal biodiversity associated with the medicinal plant *Paris* sp.

## Supplementary Material

XML Treatment for Elliptolagenoidisporium

XML Treatment for Elliptolagenoidisporium paris

## Additional information

### Use of AI

No use of AI was reported.

### Author ORCIDs

Xiang-Yu Zhang https://orcid.org/0009-0009-7330-9651

Min Liu https://orcid.org/0009-0001-8018-3980

Jian Ma https://orcid.org/0009-0008-1291-640X

Xiao-Fang Chen https://orcid.org/0009-0000-9962-7644

### Data availability

All of the data that support the findings of this study are available in the main text.
